# Supplementary material for: Shared Treg dysregulation underlies psoriasis vulgaris and atopic dermatitis through the PHF19-PRC2 epigenetic axis
Source: Front Immunol. 2026 Aug 20;17:1932788. doi: 10.3389/fimmu.2026.1932788 (PMC13538097; doi:10.3389/fimmu.2026.1932788)
Supplement: Supplementary file 3 [file DataSheet3.zip › Additional files/Additional file 8/32.IL1B.leaveoneout.pdf]

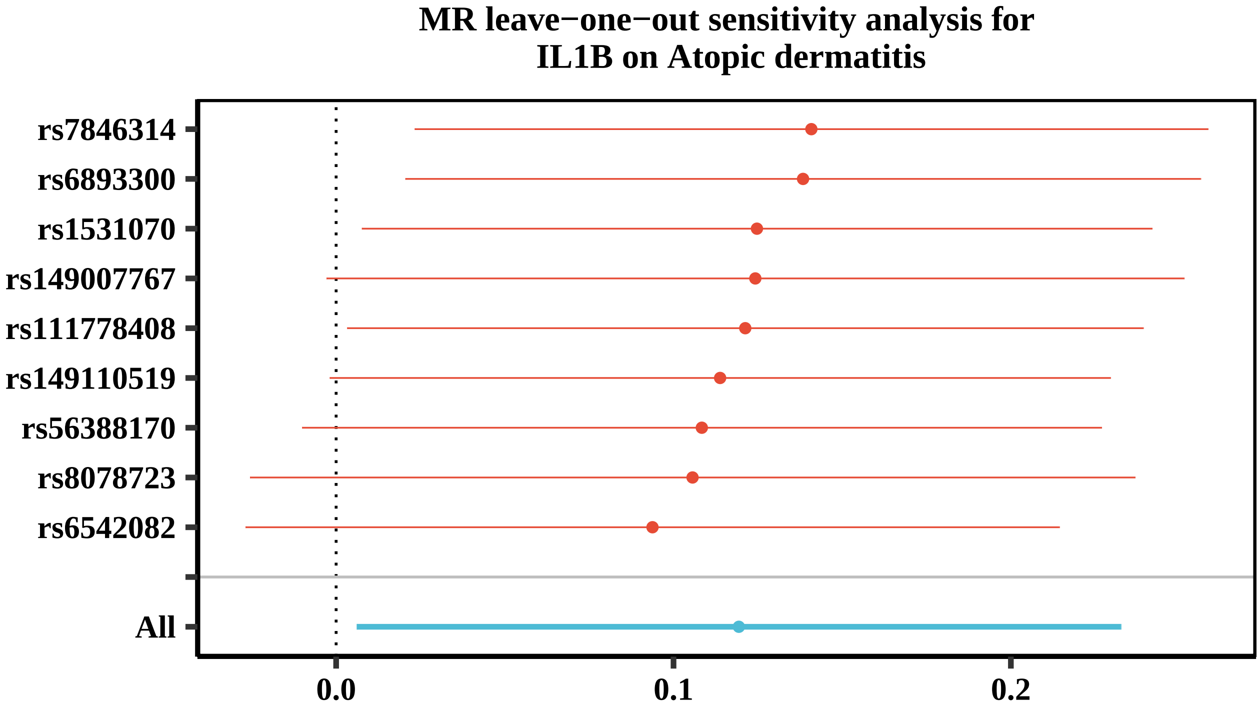

MR leave−one−out sensitivity analysis for
IL1B on Atopic dermatitis
rs7846314
rs6893300
rs1531070
rs149007767
rs111778408
rs149110519
rs56388170
rs8078723
rs6542082
All
0.0
0.1
0.2
